# Simultaneous transcranial electrical and magnetic stimulation boost gamma oscillations in the dorsolateral prefrontal cortex

**DOI:** 10.1038/s41598-022-23040-z

**Published:** 2022-11-12

**Authors:** Michele Maiella, Elias Paolo Casula, Ilaria Borghi, Martina Assogna, Alessia D’Acunto, Valentina Pezzopane, Lucia Mencarelli, Lorenzo Rocchi, Maria Concetta Pellicciari, Giacomo Koch

**Affiliations:** 1 Department of Behavioural and Clinical Neurology, Santa Lucia Foundation IRCCS, Via Ardeatina, 306, 00179 Rome, Italy; 2 Department of Psychology, La Sapienza University, Rome, Italy; 3 Center for Translational Neurophysiology of Speech and Communication, Istituto Italiano di Tecnologia (IIT), Ferrara, Italy; 4 Department of Medical Sciences and Public Health, Institute of Neurology, University of Cagliari, Cagliari, Italy; 5 Department of Neuroscience and Rehabilitation, University of Ferrara, Ferrara, Italy

**Keywords:** Neuroscience, Physiology, Neurology

## Abstract

Neural oscillations in the gamma frequency band have been identified as a fundament for synaptic plasticity dynamics and their alterations are central in various psychiatric and neurological conditions. Transcranial magnetic stimulation (TMS) and alternating electrical stimulation (tACS) may have a strong therapeutic potential by promoting gamma oscillations expression and plasticity. Here we applied intermittent theta-burst stimulation (iTBS), an established TMS protocol known to induce LTP-like cortical plasticity, simultaneously with transcranial alternating current stimulation (tACS) at either theta (θtACS) or gamma (γtACS) frequency on the dorsolateral prefrontal cortex (DLPFC). We used TMS in combination with electroencephalography (EEG) to evaluate changes in cortical activity on both left/right DLPFC and over the vertex. We found that simultaneous iTBS with γtACS but not with θtACS resulted in an enhancement of spectral gamma power, a trend in shift of individual peak frequency towards faster oscillations and an increase of local connectivity in the gamma band. Furthermore, the response to the neuromodulatory protocol, in terms of gamma oscillations and connectivity, were directly correlated with the initial level of cortical excitability. These results were specific to the DLPFC and confined locally to the site of stimulation, not being detectable in the contralateral DLPFC. We argue that the results described here could promote a new and effective method able to induce long-lasting changes in brain plasticity useful to be clinically applied to several psychiatric and neurological conditions.

## Introduction

During the last decades, a growing body of neurophysiological studies has focused on the possibility to interfere with neural oscillations. Cortical oscillatory activity represents the rhythmic activity of a population of neurons within a given frequency band^1^. Furthemore, is crucial for brain networks dynamics^2,3^ and underlies cognitive processes in the healthy^4^ and in the pathological brain^5^. Gamma oscillations in the prefrontal areas are involved in several cognitive functions including attention and memory^6,7^. Furthermore, several studies in human and animal models have suggested a role for γ oscillations in inducing and supporting synaptic plasticity mechanisms in cortical prefrontal^8^ and motor areas^9^.

Non-invasive brain stimulation (NIBS) techniques have been widely used to induce plastic changes in cortical areas, to modulate brain rhythms and influence the ongoing cortical activity. Intermittent theta-burst stimulation (iTBS) is a form of repetitive transcranial magnetic stimulation (rTMS) method, which consists of bursts of high-frequency stimulation (3 pulses at 50 Hz) repeated at intervals of 200 ms, that can induce robust long-lasting changes in the stimulated area^10^. It was firstly developed in animal models to mimic the natural patterns that support synaptic long-term potentiation (LTP) mechanisms^11,12^. iTBS has been successfully employed in the clinical ground in several conditions ranging from depression^13^ to stroke recovery^14^. These iTBS after-effects seem to be mediated by GABAergic interneurons activity, which is also crucial for oscillatory activity modulation^15–17^.

Transcranial alternating current stimulation (tACS) is another NIBS protocol delivering electrical stimulation with a sinusoid alternated current within a specific frequency. tACS can entrain ongoing brain oscillations activity and modulate brain areas in a frequency-dependent manner^18^. γtACS has been shown capable to interact with γ oscillatory activity in the primary motor cortex (M1)^19,20^ as well as in the prefrontal cortex^21^. Moreover, tACS gained attention given the therapeutic potential to induce long-lasting increases of gamma oscillations, since a decrease in gamma activity is central in various psychiatric and neurological conditions such as schizophrenia^22^ and Alzheimer’s disease^23^. However, the therapeutic effect of NIBS protocols acting on gamma oscillations is currently limited by the fact that the after-effects are often variable, small in magnitude and short-lasting^24^.

Recently, the pioneering work of Guerra and others showed that contemporary electrical and magnetic stimulation can promote robust after-effects on cortical oscillations when applied over M1^25–27^. This combination has not been assessed in areas involved in cognitive functions, such as the dorsolateral prefrontal cortex (DLPFC), which is involved in the pathophysiology of a wide range of neurological diseases^28–31^. This area is also crucial in several high-level cognition functions, such as working memory, and seems to be highly responsive to neuromodulatory protocols^32^ and pharmacological therapies^33^ when tested with TMS-EEG, as we previously demonstrated. With the premises, we targeted this area with the combined iTBS–tACS approach to understand whether this protocol can induce neuromodulatory after-effects, in particular in the gamma oscillatory activity.

One of the novelties of the present study lies in the novel approach we used to assess the effects of iTBS–tACS. Specifically, we simultaneously applied transcranial magnetic stimulation (TMS) and electroencephalography (EEG) to directly assess the cortical oscillations of a specific brain area, and so to monitor possible changes induced by the tACS-iTBS protocol. The focal perturbation of a specific area with TMS during an EEG recording allows to directly assess the natural frequencies occurring in that specific area^34,35^. In contrast, spontaneous EEG rhythms are susceptible to variability and not optimally tuned to record changes in oscillatory activity from specific areas^34,36^. We used TMS-EEG before (T0), right after the iTBS–tACS (T1) and one 20 min after (T2) the neuromodulation protocol, the last one being our target time point given that iTBS exerts its main effects after 15–20 min from its application^10,37–39^. We decided to assess the cortical oscillations with TMS-EEG because this method demonstrated to be a powerful tool to interact with the ongoing frequency of a stimulated area.

We hypothesize that the entrainment in γ frequency by tACS during iTBS could boost the long-lasting plasticity after-effects of iTBS on oscillatory activity by inducing a synergistic effect on the underlying local networks.

## Results

All 13 participants completed successfully the three-session protocol planned. The different stimulation protocols were all well-tolerated. To assess the presence of side effects or discomfort during the stimulation all the participants have to fill in a questionnaire at the end of each experimental session^40,41^. No one reported significant side effects connected with the neuromodulation protocol applications.

### Cortical oscillations results

**Figure 1:**
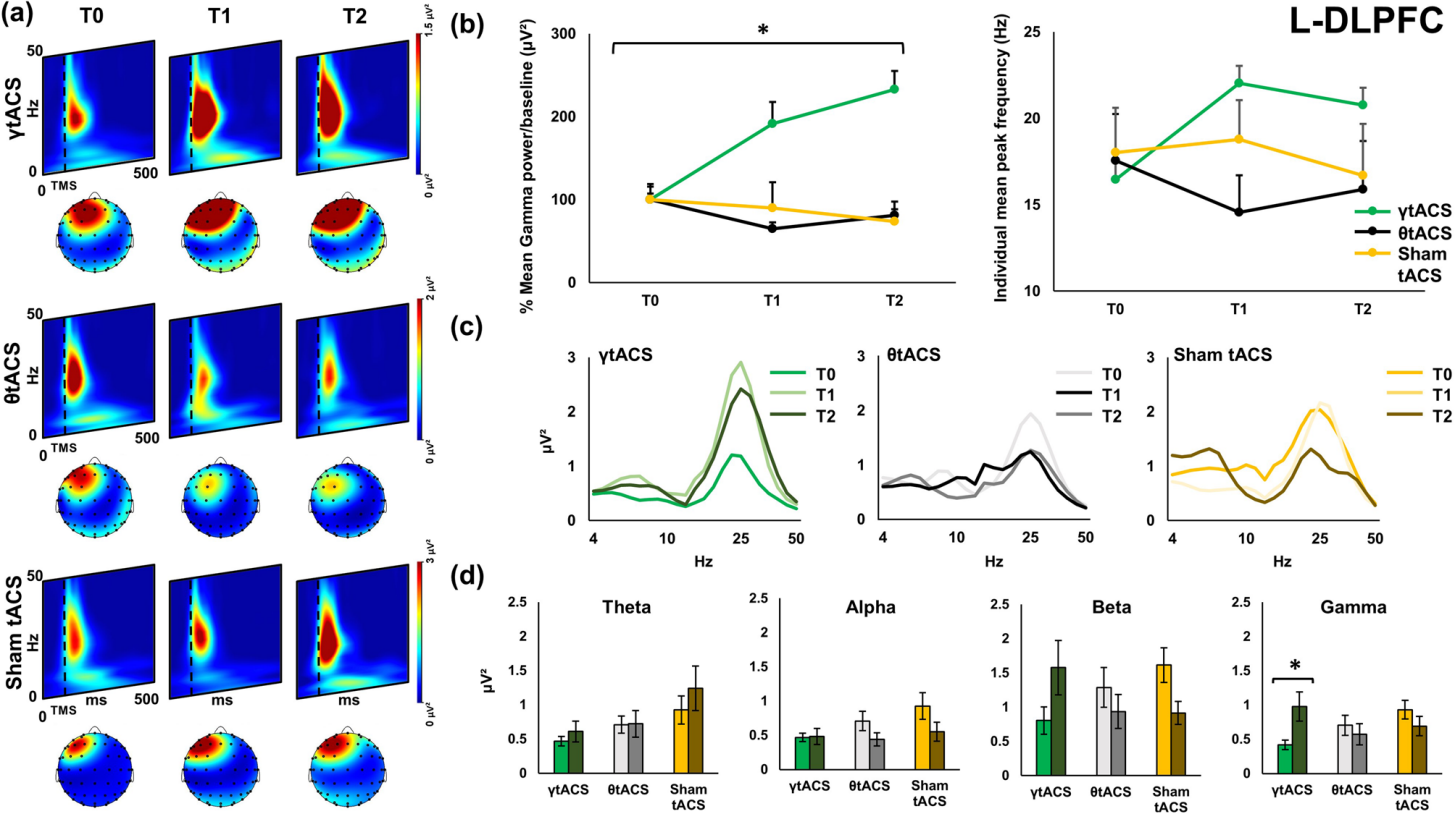
Local transcranial magnetic stimulation (TMS)-evoked cortical oscillations of the left dorsolateral prefrontal cortex (l-DLPFC). Panel (**a**) represents time–frequency oscillations for the three stimulation conditions and time points. Topographical maps depict the spatial cortical activation during TMS-EEG (50 ms after TMS pulse, 25 Hz). All maps and time–frequency representations were generated by BrainVision Analyzer (v 2.2; https://www.brainproducts.com/solutions/analyzer/). Panel (**b**) shows the power and shifting analysis performed on cortical oscillations. The panel represents on the left the % changes of the Gamma frequency in terms of power with respect to baseline. Moreover, the right side of the panel shows the shifting of the individual frequency in terms of Hz with respect to the three time points. Furthermore, panel (**c**) shows power expression of the whole frequency spectrum calculated for the cortical oscillations analysis. Lastly, panel (**d**) shows the four frequency bands considered for the analysis (Theta, Alpha, Beta, Gamma) and their change in power (μV^2^) during time (T0, T1, T2) for every stimulation condition.

**Figure 2:**
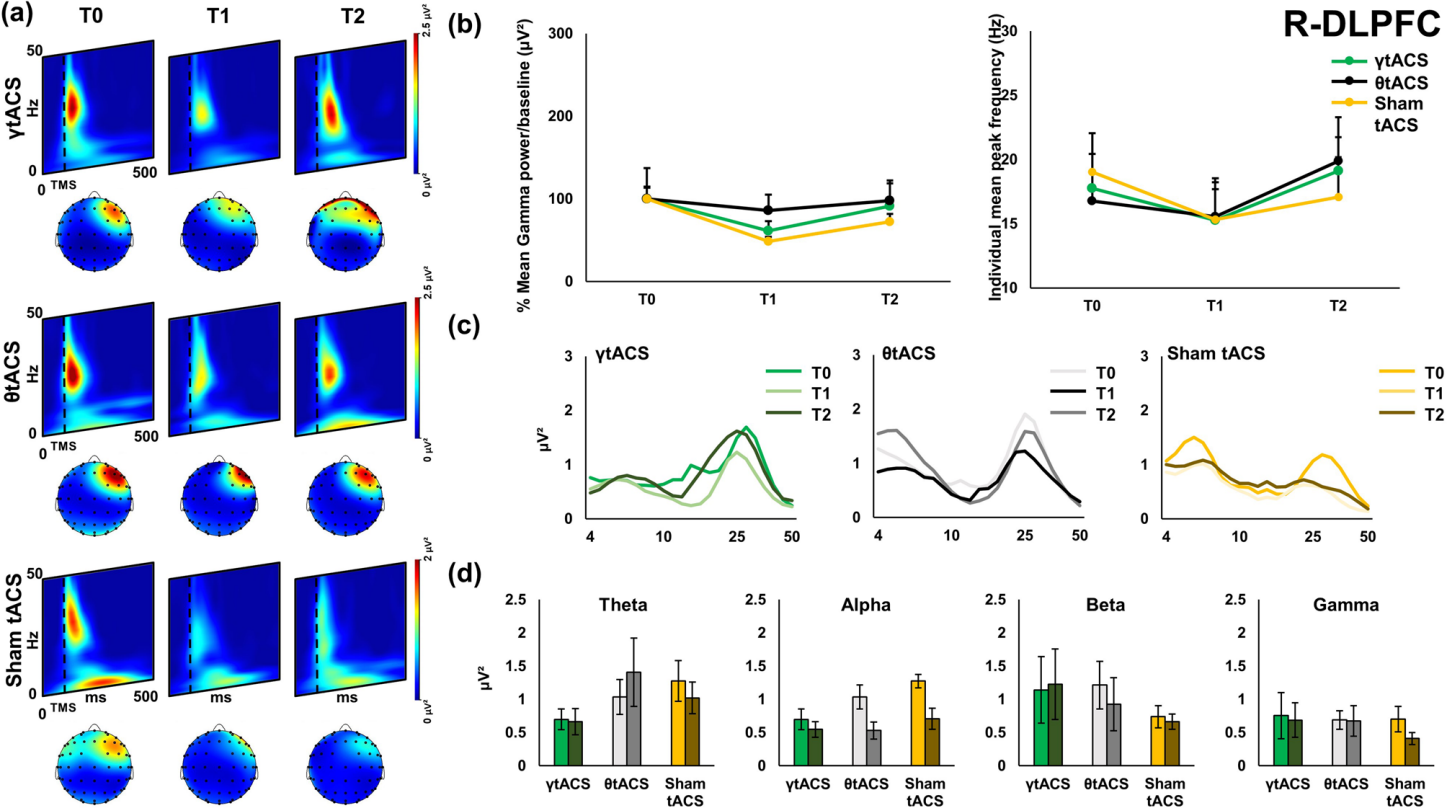
Local transcranial magnetic stimulation (TMS)-evoked cortical oscillations of the right dorsolateral prefrontal cortex (r-DLPFC). Panel (**a**) represents time–frequency oscillations for the three stimulation conditions and time points. Topographical maps depict the spatial cortical activation during TMS-EEG (50 ms after TMS pulse, 25 Hz). All maps and time–frequency representations were generated by BrainVision Analyzer (v 2.2; https://www.brainproducts.com/solutions/analyzer/). Panel (**b**) shows the power and shifting analysis performed on cortical oscillations. The panel represents on the left the % changes of the Gamma frequency in terms of power with respect to baseline. Moreover, the right side of the panel shows the shifting of the individual frequency in terms of Hz with respect to the three time points. Furthermore, panel (**c**) shows power expression of the whole frequency spectrum calculated for the cortical oscillations analysis. Lastly, panel (**d**) shows the four frequency bands considered for the analysis (Theta, Alpha, Beta, Gamma) and their change in power (μV^2^) during time (T0, T1, T2) for every stimulation condition.

**Figure 3:**
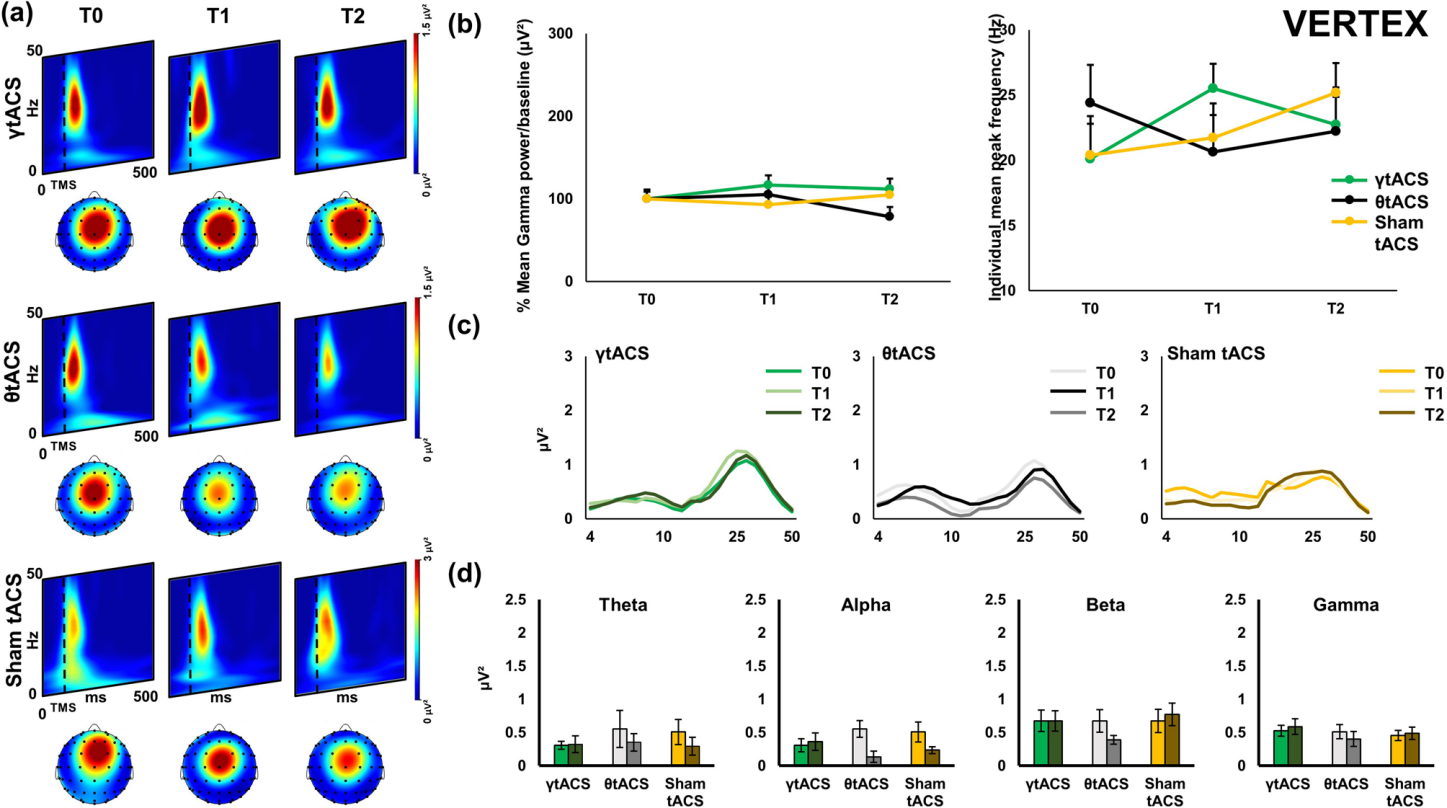
Local transcranial magnetic stimulation (TMS)-evoked cortical oscillations of the Vertex. Panel (**a**) represents time–frequency oscillations for the three stimulation conditions and time points. Topographical maps depict the spatial cortical activation during TMS-EEG (50 ms after TMS pulse, 25 Hz). All maps and time–frequency representations were generated by BrainVision Analyzer (v 2.2; https://www.brainproducts.com/solutions/analyzer/). Panel (**b**) shows the power and shifting analysis performed on cortical oscillations. The panel represents on the left the % changes of the Gamma frequency in terms of power with respect to baseline. Moreover, the right side of the panel shows the shifting of the individual frequency in terms of Hz with respect to the three time points. Furthermore, panel (**c**) shows power expression of the whole frequency spectrum calculated for the cortical oscillations analysis. Lastly, panel (**d**) shows the four frequency bands considered for the analysis (Theta, Alpha, Beta, Gamma) and their change in power (μV^2^) during time (T0, T1, T2) for every stimulation condition.

Figures 1, 2 and 3 show local TMS-evoked local oscillatory activity for the three areas assessed with TMS-EEG: left dorsolateral prefrontal cortex (l-DLPFC; Fig. 1); right dorsolateral prefrontal cortex (r-DLPFC; Fig. 2); and the vertex (Fig. 3). As depicted in the wavelets representations of the three figures (panels (a)), TMS-evoked cortical oscillations have similar baseline activity patterns characterized by a remarkable activation around 50 ms after TMS pulse in the frequencies between 20 and 30 Hz. A second activation can be identified from around 50 ms to 250 ms in lower frequencies such as approximately 6 Hz to 10 Hz. Figure 1 panel (b) shows power and shifting analysis of local oscillatory activity of l-DLPFC. Gamma oscillation power analysis shows a significant Time × tACS interaction [F(2,24) = 4.923, *p* = 0.016, η^2^ = 0.291]; post-hoc analysis reveals that after iTBS–γtACS the power in gamma frequency was significantly enhanced [post-hoc *p* = 0.017]. No effects were observable after the other tACS factors (iTBS–θtACS [F(2,24) = 0.152, *p* = 0.860, η^2^ = 0.012], sham-tACS [F(2,24) = 0.699, *p* = 0.507, η^2^ = 0.055]). Figure 1 panel (b) focuses in the right side on the individual mean peak frequency shifting right after the iTBS–tACS neuromodulation protocol, a repeated-measures ANOVA shows a trend for a significant Time × tACS interaction in shifting the most expressed individual mean peak frequency [F(2,24) = 3.335, *p* = 0.053, η^2^ = 0.217]; post-hoc analysis reveal a significant difference in γtACS factor between T0 vs T1 Time points [*p* = 0.044] but not in θtACS [*p* = 0.207] and sham tACS [*p* = 0.591] factors. Panel (c) shows power expression of the whole frequency spectrum calculated for the cortical oscillations analysis. Panel (d) depict the four frequency bands considered for the analysis (theta, alpha, beta, gamma) and their change in power before and after (T0 vs T2) each iTBS–tACS protocol (iTBS–γtACS/θtACS/sham tACS) for each stimulation condition. Figures 2 and 3 show the results of the other two areas considered for the analysis. No significant effects were found for both of them (all ps > 0.05). Bayesian statistical analysis (RM-ANOVA) on power indicated that there is weak support for models that include main effects of factors “tACS” (BF10 = 0.187), “time” (BF10 = 0.263), “tACS + time” (BF10 = 0.046) and “tACS + time + tACS × time” (BF10 = 0.177). In fact, all considered models support the null hypothesis. Among post hoc comparisons, only the comparison between T0 and T2 in the γ tACS condition was slightly suggestive of the alternative hypothesis (BF10 = 3.765). The Bayesian (RM-ANOVA) on PLV yielded very strong evidence in favour of the alternative hypothesis. In detail, support was given from the main effect of factor “electrode” (BF10 = 92,320.361), “electrode + time” (BF10 = 15,289.236) and, most importantly, “electrode + time + electrode × time” (BF10 = 2.578e+6). Very strong support to the alternative hypothesis was also given by post-hoc comparisons of T0 vs T2 for both electrodes F5 (BF10 = 30.649) and F2 (BF10 = 46.98).

**Figure 4:**
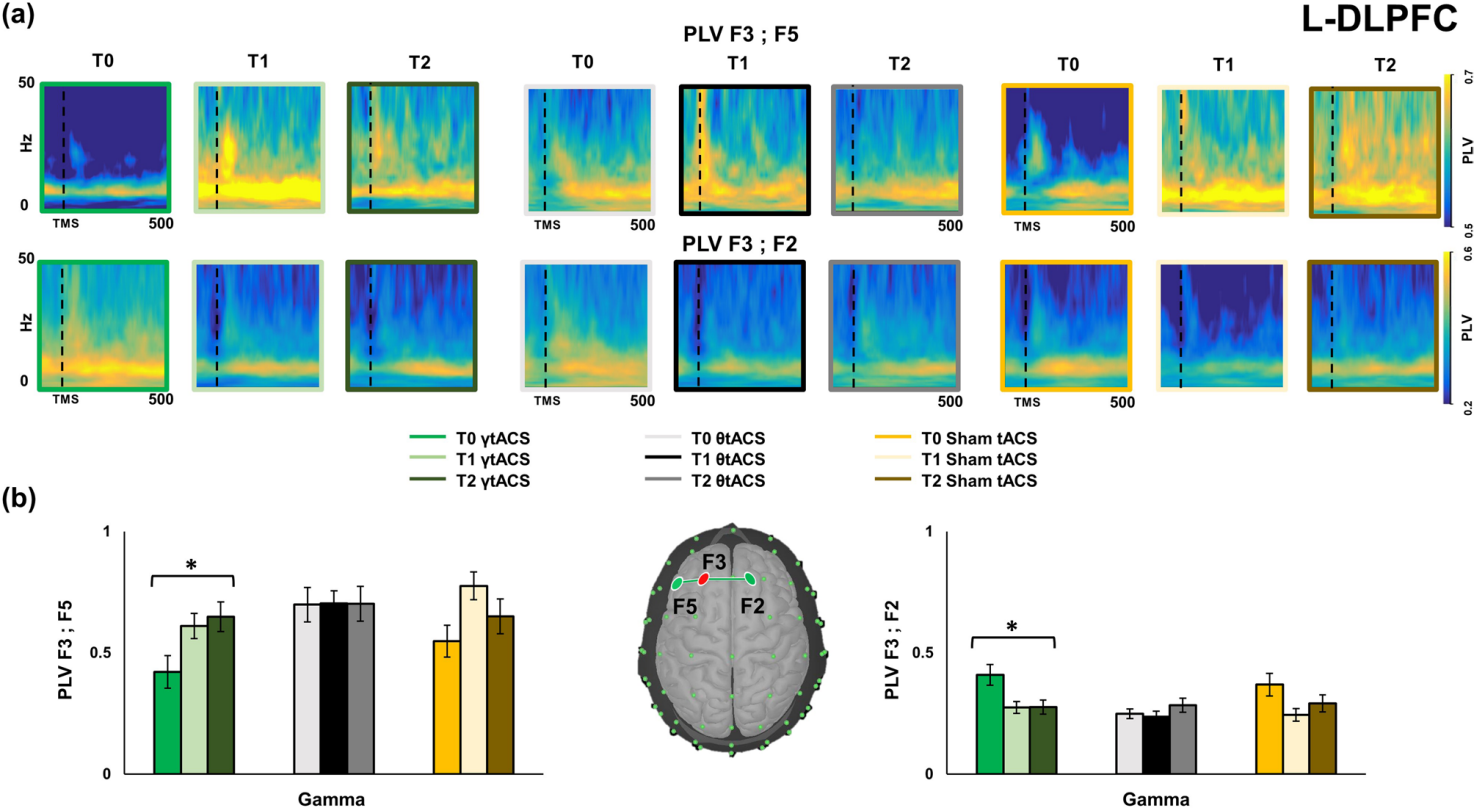
Paired electrodes Wavelet Phase-locking value (W-PLV) for the three stimulation conditions and time points on l-DLPFC. Panel (**a**) shows the W-PLV in a frequency (Hz) for time (ms) representation for each stimulation and time condition in both the paired electrodes considered for the analysis (F3 vs F5; F3 vs F2). All W-PLV time–frequency representations were generated by BrainVision Analyzer (v 2.2; https://www.brainproducts.com/solutions/analyzer/). Panel (**b**) shows the histograms for the W-PLV in Gamma frequency for both the paired electrodes considered for the analysis. In the centre, a topographical representation of the electrodes is coupled for the analysis.

Figure 4 shows the individual frequency wavelet phase-locking value analysis (W-PLV). W-PLV was conducted on the data collected during l-DLPFC TMS-EEG recordings between F3-F5 and F3-F2 before and after iTBS–tACS for the three tACS conditions. Panel (a) represents W-PLV in F3-F5 and F3-F2 channels pairs for the tACS conditions in time. PLV values are higher in T0 for lower frequency bands and in a range between 22 and 28 Hz for both couples of electrodes. Figure 4 panel (b) represents histograms that show the neuromodulation iTBS–γtACS effects on W-PLV in l-DLPFC during time (T0, T1, T2) for a mean gamma band range (30–50 Hz). W-PLV analysis shows a significant interaction between factors Electrode × Time [F(2,22) = 20.817, *p* = 0.01, η^2^ = 0.654] for the Gamma frequency (mean between 30 and 50 Hz), post-hoc analysis shows significant difference between T0 and T1 [F5 *p* = 0.05, F2 *p* = 0.01] and T0 and T2 [F5 *p* = 0.004, F2 *p* = 0.002] for both the F3-F5 and the F3-F2 pairs. These effects are depicted respectively in panel (b)’s left (F3-F5) and right (F3-F2) side histograms. No significant effects were reported for the same analysis in r-DLPFC and vertex.

### Cortical excitability results

**Figure 5:**
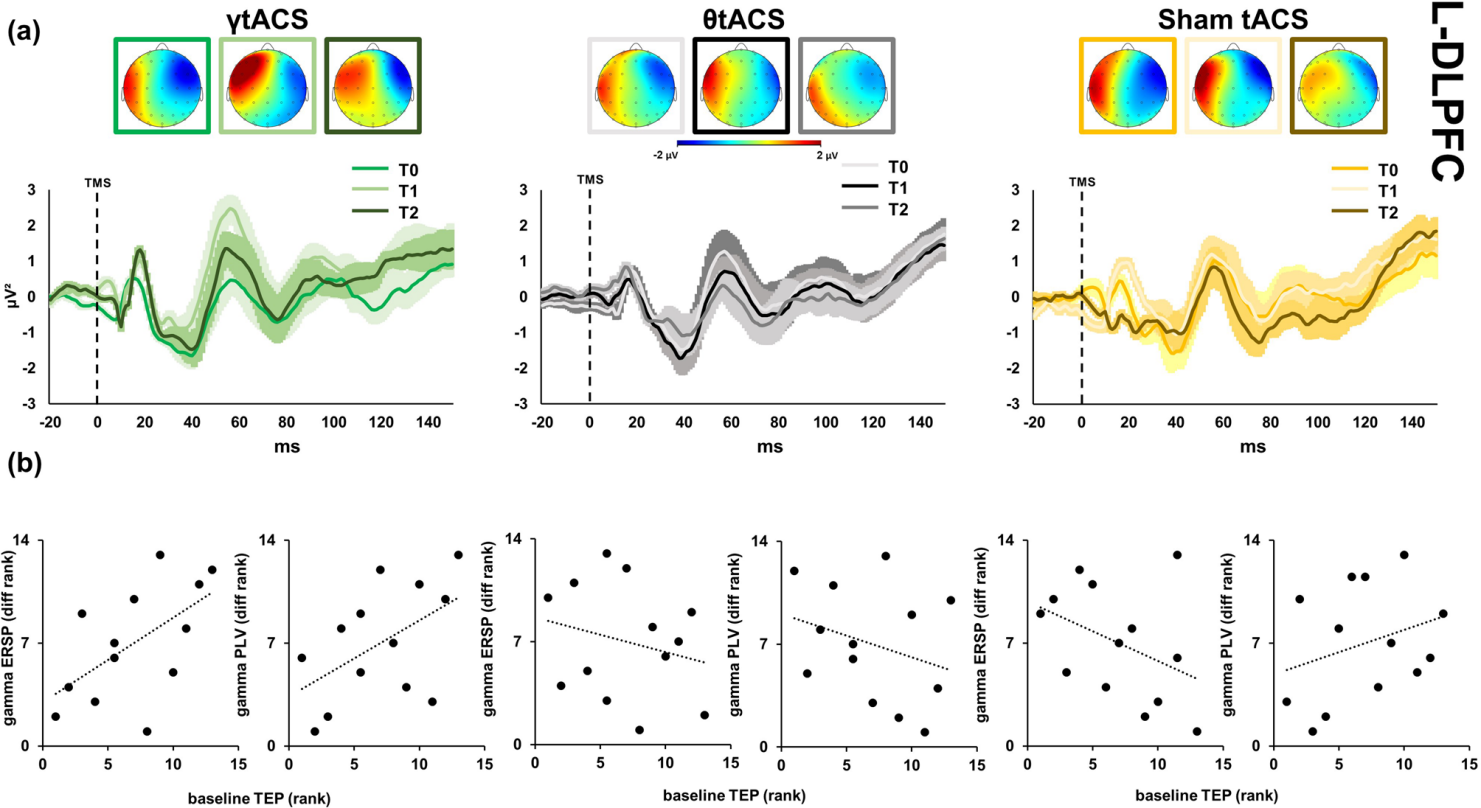
Local transcranial magnetic stimulation (TMS)-evoked cortical response of left dorsolateral prefrontal cortex (l-DLPFC). Left side graphics depict TMS-evoked activity after iTBS–γtACS stimulation (green colours), centre graphics after iTBS–θtACS stimulation (black colours) and right side graphics after iTBS-sham tACS stimulation (yellow colours). Top maps (panel (**a**)) represent the topographic activity within the third calculated peak (from 48 to 65 ms; − 2 μV to 2 μV amplitude) in three times (from left to right respectively T0, T1, T2 time points). Furthermore, panel (**a**) shows TMS-EEG cortical response before (T0), right after (T1) and 20 min after stimulation (T2). All maps were generated by BrainVision Analyzer (v 2.2; https://www.brainproducts.com/solutions/analyzer/). Panel (**b**) depicts correlation graphics between the baseline TEPs amplitude, ERSP and PLV for the three tACS conditions.

**Figure 6:**
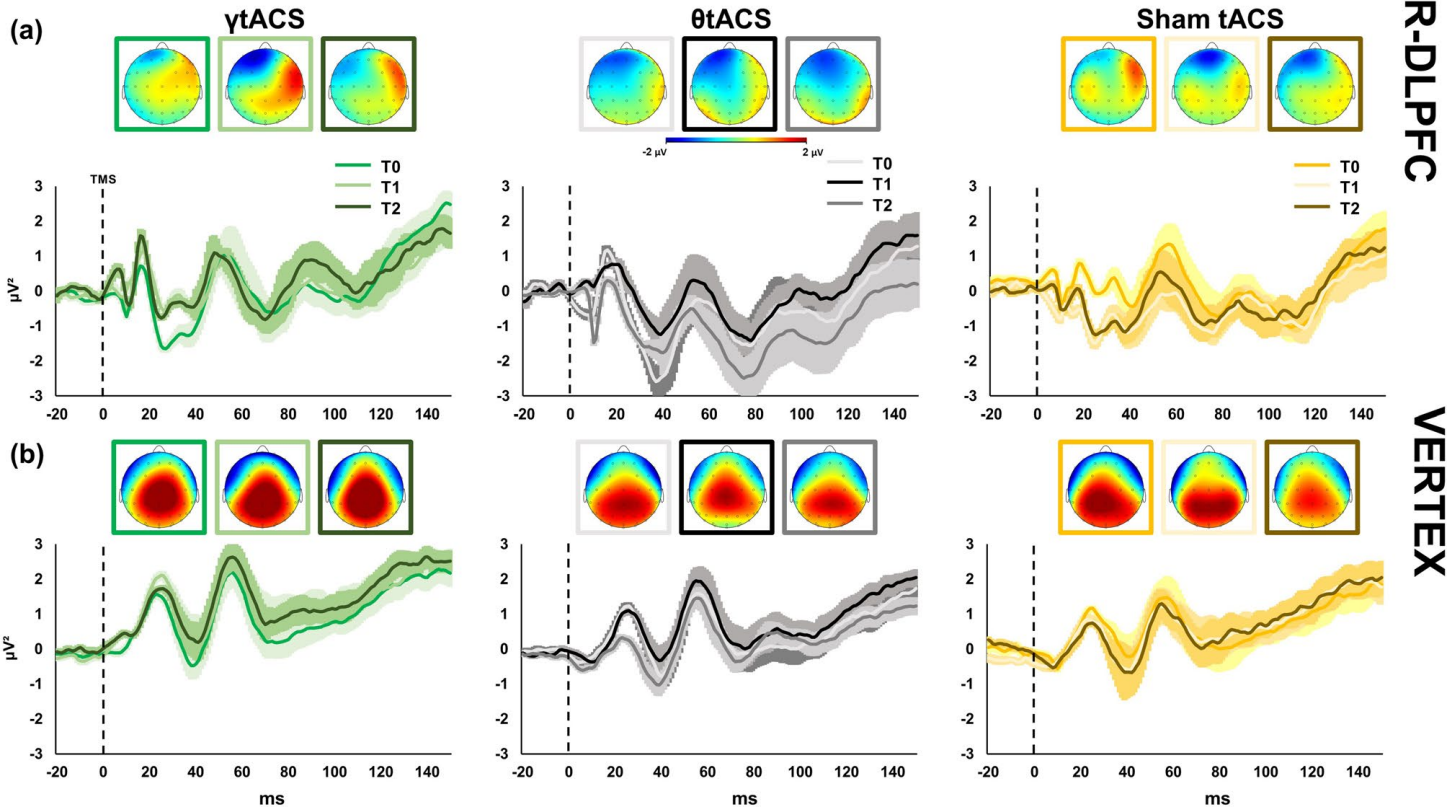
Local transcranial magnetic stimulation (TMS)-evoked cortical response of left dorsolateral prefrontal cortex (l-DLPFC, panel (**a**)) and Vertex (panel (**b**)). Left side graphics depict TMS-evoked activity after iTBS–γtACS stimulation (green colours), centre graphics after iTBS–θtACS stimulation (black colours) and right side graphics after iTBS–sham tACS stimulation (yellow colours). Top maps (panel (**a**, **b**)) represent the topographic activity within the third calculated peak (from 48 to 65 ms; − 2 to 2 μV amplitude) in three times (from left to right respectively T0, T1, T2 time points). Panel (**a**, **b**) shows TMS-EEG cortical response before (T0) right after (T1) and 20 min after stimulation (T2). All maps were generated by BrainVision Analyzer (v 2.2; https://www.brainproducts.com/solutions/analyzer/).

**Figure 7:**
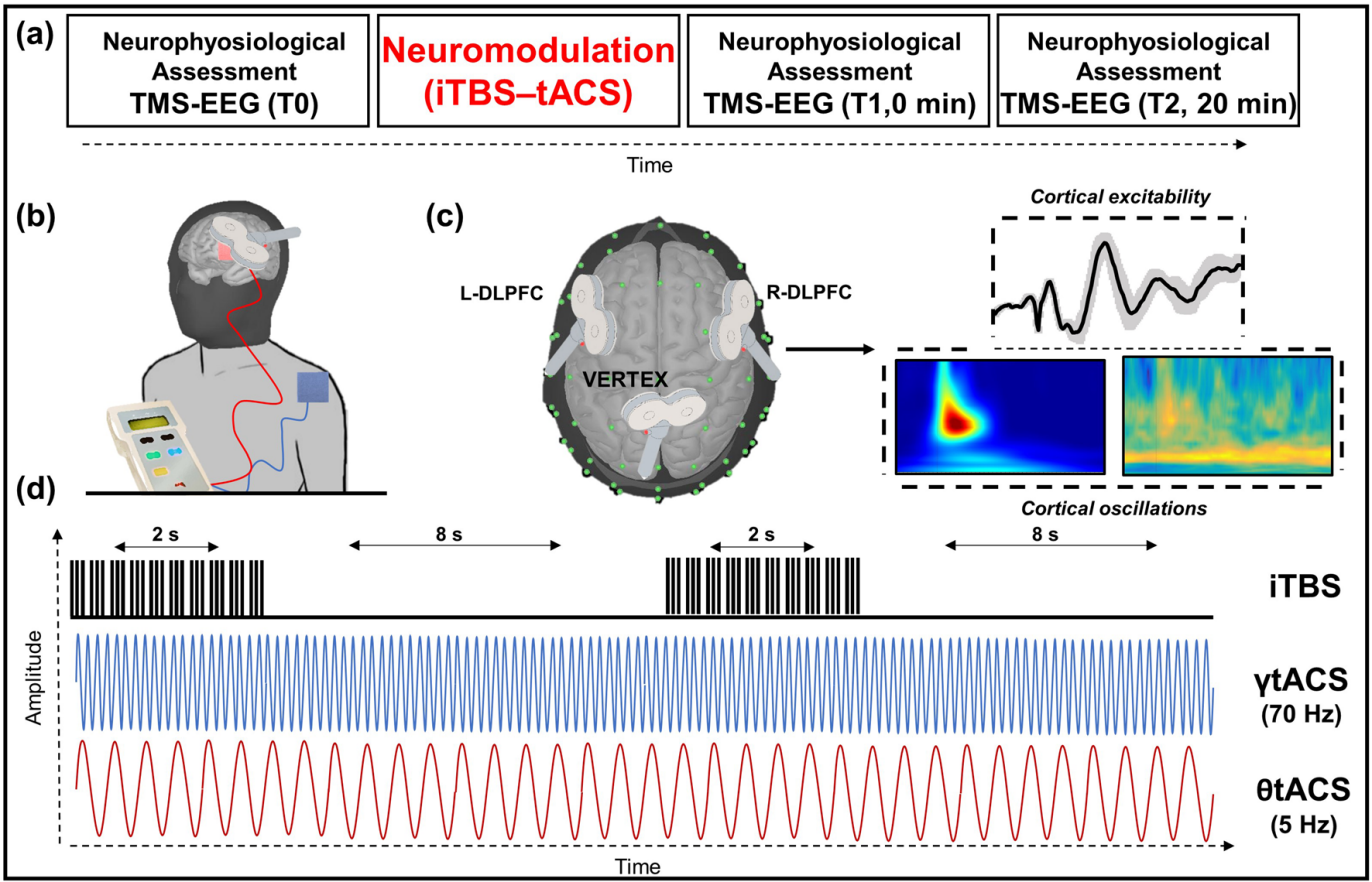
(**a**) Single experimental session design; iTBS–tACS neuromodulation effects were assessed before, at 0 min, and 20 after the stimulation. (**b**) Focus on the iTBS–tACS on left dorsolateral prefrontal cortex (l-DLPFC). (**c**) Focus on the three sites stimulated to assess the effects of stimulations with TMS-EEG. Next to the sites figures are an example of domains interested by the analysis. (**d**) Stimulation protocols example for intermittent theta burst stimulation (iTBS) and both gamma (γ) and theta (θ) transcranial alternated current stimulation (tACS). iTBS delivered triplets of magnetic pulses that last for 2 s with an interval of 10 s between stimulation trains; tACS delivered electrical stimulation waves at 70 Hz or 5 Hz depending on the experimental session.

Single-pulse TMS evoked on the EEG signal a well-known sequence of positive and negative deflections with amplitude ranging from − 3 to 3 μV and lasting up to ~ 250 ms (Fig. 5). TMS-evoked cortical activity (Figs. 5, 6, 7) last around 150 ms and it is characterized by a series of peaks such as P1 from 15 to 25 ms, P2 from 26 to 47 ms, P3 from 48 to 65 ms, P4 from 66 to 75 ms, P5 from 76 to 115 ms, P6 from 116 to 145 ms. This temporal dynamic, in terms of waveform and amplitude, looks similar for all the sites at baseline. The three peaks were detectable over all the three stimulated areas as expected^32,34^. No differences in the general amplitude are detected between l-DLPFC, r-DLPFC and Vertex. In the first two peaks window (i.e. 15–60 ms after TMS) a dipole was focused over the stimulated area; from ~ 65 to ~ 120 ms after TMS spread negativity observable over both the hemispheres, followed by a strong positivity centred over the frontocentral electrodes, ranging from ~ 120 to ~ 250 ms after TMS. Figure 3 shows the TMS-evoked potential (TEPs) over the different stimulation sites pooling (F1, F3, FC1) for l-DLPFC in tACS and time factors.

### Cortical excitability and oscillations correlations results

Correlation analyses (Fig. 5 panel (b)) showed a direct linear relationship between the baseline TEP amplitude and the observed differences in ERSP (r_s_ = 0.575, *p* = 0.02) and PLV (r_s_ = 0.517, *p* = 0.035), specifically for γtACS condition. When the same analysis was performed for the θtACS and sham-tACS conditions, we did not observe any significant relationship (all ps > 0.05).

Regression analysis showed that the baseline level of excitability, as measured with TEP amplitude at T0, was a significant predictor of the iTBS–γtACS effects in cortico-cortical connectivity, as measured with PLV [R^2^ = 0.319, β = 0.045, *p* = 0.044].

## Discussion

Here we show that the simultaneous application of iTBS–gamma-tACS exerts a robust long-lasting increase in gamma oscillations when applied over the l-DLPFC. Specifically, the enhancement in gamma activity was visible over different physiological measures: on the power oscillation frequency analysis and in the local connectivity. These results were specifically observable when tested over the stimulated l-DLPFC, not being detectable in the contralateral DLPFC nor in the vertex.

One of the aims of this study was to test the potential of the combined iTBS–tACS approach to produce robust long-lasting oscillatory brain changes on DLPFC and to evaluate whether a synergistic effect would arise from this combination. Indeed, although there is great interest in the application of iTBS in several neurological and psychiatric disorders, its clinical impact is somewhat limited by the variability of after-effects that have been reported in healthy studies evaluating the effects on the amplitude of the MEP^39^. These studies showed that only approximately 50% of participants undergoing iTBS show the expected significant motor MEP long-lasting increase^42–45^. Inter-subject variability is considered the most relevant limitation of non-invasive brain stimulation^46,47^ since it affects the effectiveness of neuromodulation techniques and limits their clinical applications. Moreover, the magnitude of increase in MEPs amplitude after M1 iTBS and in TEPs amplitude after DLPFC iTBS is in the range of 20–50% as compared to baseline, again potentially limiting the clinical impact of this plasticity inducing protocol^48^. While initially iTBS was thought to produce more powerful and reproducible effects than other rTMS methods, a claim that has not been fully confirmed, its main attraction still relies on the speed of application with protocols lasting a few hundreds of seconds instead of several minutes^38,48,49^. Indeed, iTBS through the high-frequency neuronal activation modulates cortical inhibition and GABA-ergic synaptic transmission, resulting in an enhancement of γ band expression^50^. iTBS is thought to activate Ca^2−^ influx to the postsynaptic neuron. The property, including the amount and the rate of the increase, determines the amount of the build-up of subsequent facilitation processes that modify the synaptic strength^51^. This notion is supported by animal studies showing that dysfunction of Inositol 1,4,5-trisphosphate receptors (InsP3Rs) is required for conversion of LTD to LTP, while partial blockade of NMDARs to reduce the rate of Ca^2−^ influx, results in a conversion of LTP to LTD^52^.

On the other hand, tACS is a relatively new technique that can effectively modulate oscillatory brain activity through weak external alternating current at specific frequencies^18^. This effect is supported by research in animals^53^ suggesting that transcranial electrical stimulation (tES) in phase with network-induced patterns can enhance neuronal discharge activity.The electric field, which in itself may be subthreshold, can be effectively summed with otherwise subthreshold effects of network-induced membrane voltage fluctuations. This combined effect can generate spikes in a fraction of the neuronal population through the mechanism of stochastic resonance^54,55^. However, there is still a lack of understanding on the exact mechanisms that modulate cortical activity as a function of tACS administration.

We argue that the robust synergistic effects observed here are the consequence of the interplay among gamma oscillations and the formation of cortical plasticity. The role of gamma activity in synaptic plasticity has been then confirmed throughout the last two decades by numerous investigations using electrophysiological recordings in animals^56^ and humans^36,57^. Although the exact physiological mechanism is still a matter of debate, it has been suggested that local inhibitory interneurons play a key role in synchronizing gamma oscillations among large neuronal populations^58–61^. Thus, when depolarized, local interneuron populations tend to generate synchronized inhibitory postsynaptic potentials in thousands of cells, inducing an entraining in fast gamma oscillations not only in local but also in distant neurons^32,59,62,63^. Interstingly, our results show a direct linear relationship between the initial level of cortical excitability in the l-DLPFC and in the physiological responses to the neuromodulatory protocol, in terms of connectivity and power improvement. Specifically, higher baseline level in cortical excitability resulted in a stronger enhancement of gamma oscillations and gamma-mediated connectivity. Again, this effect was specifically observable for the target gamma-tACS over the l-DLPFC, probably due to the capacity of this associative area to involve local GABA-ergic interneuron populations thus resulting in a stronger changes in local plasticity and network connectivity changes^32,64,65^. This is relevant since gamma-frequency synchronization between the activity of distant neuronal cells has emerged as a marker of connectivity within large cortical networks, during learning or memory processing^66,67^. During the formation of plasticity, an increase in gamma-activity coherence represents enhanced connectivity between distant neuronal populations in forming a new memory^68^. This latter element is particularly relevant since cognitive dysfunction in AD has been recently linked to a disorder of gamma oscillations^23,36^. In AD animal models, local changes in gamma oscillatory activity affect multiple brain centres critical for learning and memory, and other higher-order brain functions, such as the hippocampus and the prefrontal cortex^69–73^. Hence we believe that our current findings may have broad implications for treating gamma dysregulation in neurodegenerative disorders such as AD.

While we found that combined iTBS–γtACS induced robust after-effect on DLPFC cortical activity both in terms of excitability and oscillations, combined iTBS–θtACS did not result in any significant change. This finding is in line with the pioneering work of Guerra and colleagues showing that tACS delivered at lower frequencies in the alpha band leaves the iTBS-related LTP-like plasticity unchanged^25,26^.

The study has some limitations. Firstly, delivering θtACS and γtACS on l-DLPFC without simultaneous iTBS could be adequate to control the effects of θ- and γtACS–iTBS. However, it has been shown previously that a few seconds of tACS are not supposed to exert any after-effect^74^ and thus we did not weigh down our experimental procedure which was already quite demanding for the healthy participants recruited for the study. The TMS-EEG measurements did not include a sham stimulation condition to control for peripherally evoked potentials and muscle artefacts. In this regard, we adopted several methodological precautions to avoid auditory and somatosensory artifacts that can affect the EEG response^75^. To reduce the auditory response, we used an ad-hoc masking noise; to reduce bone conduction of the TMS click and scalp sensation caused by coil vibration we placed a 0.5 cm foam layer underneath the coil. It is also important to note that in the present study we did not deliver TMS basing on the tACS phase using a closed-loop setup. Other approaches are trying to apply controllable phase-synchronized rTMS with tACS to induce and stabilize neuro-oscillatory resting-state activity at targeted frequencies. For instance, Hosseinian and colleagues^76^ used a novel circuit to precisely synchronize rTMS pulses with the phase of tACS in the bilateral prefrontal cortex (PFC). They found that 10-Hz resting-state PFC power increased significantly with peak-synchronized rTMS-tACS, while rTMS timed to the negative tACS trough did not induce local or global changes in oscillations. Moreover, they also developed a novel stimulation protocol, where a single circuit precisely synchronizes rTMS pulses with the phase of tACS in the theta frequency band^77^. Similarly, Zrenner and colleagues^78^ hypothesized that triggering TMS synchronized with the negative peak of endogenous alpha oscillations in left DLPFC would more effectively increase cortical excitability (as measured with TMS-evoked potentials) than a non-alpha-synchronized stimulation protocol. Finally, while our evidence seems to suggest that the synchronization of rTMS with peak oscillatory activity may have an impact on subsequent plasticity induction, this approach is technically limited to lower frequency bands in the theta-alpha range. Current methodological restraints do not allow to transfer of a similar approach towards higher frequencies such as those used in the gamma band in our case since these cannot be reliably detected online with non-invasive scalp EEG recordings. Such hypothesis however could be tested in the future in patients with implanted electrodes.

The results of the present study are relevant both from a neurophysiological point of view and in a clinical perspective. On one hand, we showed that tACS is able to boost the effects of iTBS on the modulation of GABA-ergic synaptic transmission and to interact with γ activity mediating cortical plasticity in l-DLPFC. Future studies have to focus on the nature of this connection and its neurophysiological mechanisms. From a clinical perspective, the observed changes in the oscillatory activity and connectivity dynamics after the iTBS–γtACS application, could shed light in the development of novel comhined NIBS protocols useful in the treatment of several psychiatric and neurological conditions.

## Methods

### Participants and procedure

13 healthy participants (7 females, mean 27.6 years, SD ± 2.5) were enrolled in the study. All the participants were recruited from a list of healthy individuals available for the researchers of IRCCS Santa Lucia Foundation. They underwent the study for free and didn’t receive any payment in any form (money, university credit or similar). This was an experimental within-subject design including three different randomized sessions for each participant. During every session, participants underwent a combined neuromodulatory protocol with iTBS and tACS over the l-DLPFC. We used three different tACS protocols for each session: (1) iTBS–γtACS; (2) iTBS–θtACS; (3) iTBS–sham tACS. To investigate the effects of the neuromodulatory protocol, we used single-pulse TMS combined with EEG recordings before (T0) iTBS–tACS, 0 min (T1) and 20 min (T2) after the iTBS–tACS protocol. TMS-EEG was applied over three cortical areas: l-DLPFC, r-DLPFC and vertex. The order of stimulation of the three areas was randomized. To make sure no long-lasting effects influence experimental results, sessions were separated by at least one week one from another. The study was approved by the local ethics committee (Ethical Committee Fondazione Santa Lucia; Prot. CE/PROG.811; 19/02/2021). TMS safety guidelines and medical regulations were fully followed by experimenters and written informed consent was obtained from each participant.

### iTBS–tACS neuromodulation protocol

We applied a combined iTBS–tACS protocol based on previous studies conducted by Guerra and colleagues^26^.

The iTBS–tACS was applied over l-DLPFC and last for 190 s, with the tACS electrode on the scalp and the iTBS coil just above it. A figure-of-eight coil with a diameter of 70 mm was used to deliver iTBS over the scalp site corresponding to the l-DLPFC (F3, according to 10–20 system). A MagStim Rapid^2^ magnetic stimulator (Magstim Company, Whitland, Wales, UK) was used to deliver the biphasic waveform pulse, with a pulse width of ∼ 0.1 ms. iTBS consist of a 2-s train of TBS that is repeated 20 times, every 10 s for a total of 190 s (600 pulses). Stimulation intensity was set at 80% of the active motor threshold (AMT), defined as the lowest intensity able to produce MEPs < 200 μV in at least five out of ten trials when the participant performed a 10% of maximum contraction using visual feedback^79^. AMT was tested over the motor cortex of the target hemisphere of iTBS (i.e. left) with the same stimulation condition, i.e. with the tACS electrode under the coil. Electromyographic activity was recorded from the contralateral FDI muscle, using two Ag–AgCl surface cup electrodes (9 mm) in a belly-tendon montage. Responses were amplified through a Digitimer D360 amplifier (Digitimer Ltd, Welwyn Garden City, Hertfordshire, UK): filters were set at 20 Hz and 2 kHz, with a sampling rate of 5 kHz; they were then recorded by a computer using SIGNAL software (Cambridge Electronic Devices, Cambridge, UK). During stimulation, the coil was oriented 45° with respect to l-DLPFC (F3). A neuronavigation system (SofTaxic; E.M. S., Bologna s.r.l.) coupled with a Polaris Vicra infrared camera was used to ensure that in each participant iTBS was applied over the same spot across different sessions. Indeed, stimulation point was sampled in the neuronavigation system during the first session and imported in the following two sessions. A Brainstim multifunctional system for low-intensity transcranial electrical stimulation (E.M.S., Bologna s.r.l.) was used to deliver the current stimulation using saline‐soaked sponge electrodes (7 × 5 cm^2^). The active electrode (anode) was placed on the scalp over the l-DLPFC and the reference (cathode) over the right deltoid muscle. During real stimulations, the current was set to 1 mA and delivered for 190s^26^. For the sham condition, the electric current was not applied, but there were a 2 s 1 mA ramp up and 2 s 1 mA ramp down, to give the participant real stimulation feelings. For γtACS the sinusoid frequency wave was set at 70 Hz; for θtACS, the sinusoid frequency wave was set at 5 Hz. iTBS and tACS were synchronized using a BrainTrigger (E.M. S., Bologna s.r.l.) and SIGNAL Software so that both stimulations started simultaneously; consequently no ramp-up and no ramp down were programmed for the stimulation.

### TMS–EEG neurophysiological assessment

During every session, participants underwent an electroencephalographic (EEG) recording of a series of TMS pulses delivered in specific areas of interest. Consequently, it was possible to evaluate cortical reactivity, connectivity and plasticity during time. TMS pulses were applied over the l-DLPFC, r-DLPFC and vertex. These three cortical areas were assessed three times each: before (T0), right after (T1) and 20 min after (T2) neurostimulation protocol. Neurophysiological assessments on cortical areas were randomized at every time point. According to scientific literature, the coil was differently oriented with respect to the mid-sagittal axis of the participant's head for each stimulation site: 45° over l-DLPFC and r-DLPFC, and parallel over the vertex, with the handle pointing backwards. The intensity of stimulation of single-pulse TMS was set at 110% of the resting motor threshold (RMT), defined as the lowest intensity producing MEPs of > 50 μV in at least five out of 10 trials in the relaxed first dorsal interosseous (FDI) muscle of the right hand^80^. For what concern TMS devices, monitoring neuronagivation system, and others stimulation conditions were all identical to neuromodulation protocol described in the paragraph above.

A TMS‐compatible DC amplifier (BrainAmp, Brain Products GmbH, Munich, Germany) was used to record EEG activity from the scalp. The EEG was continuously recorded from 64 sites positioned according to the 10–20 International System, using TMS‐compatible Ag/AgCl pellet electrodes mounted on an elastic cap. Additional electrodes were used as ground and reference. The ground electrode was positioned in AFz, while the reference was positioned on the tip of the nose. EEG signals were digitized at a sampling rate of 5 kHz. Skin/electrode impedance was maintained below 5 kΩ. Horizontal and vertical eye movements were detected by recording the electrooculogram to offline reject the trials with ocular artefacts.

Each TMS-EEG session consisted of single pulses (100 for T0, 80 for T1 and T2) applied at a random inter-stimulus interval (ISI) of 2 s with a variation of 20%. During TMS-EEG assessment participants passive listen to a white noise and wear ear protectors to ensure the environmental noise does not affect the EEG signal. A short break was run between TMS-EEG sessions of either site. During the entire session, participants were seated on a dedicated, comfortable armchair in a soundproofed room.

### TMS–EEG analysis

TMS–EEG data were analyzed offline with Brain Vision Analyzer (Brain Products GmbH) and EEGLAB toolbox running in a MATLAB environment (MathWorks Inc., Natick, MA). As a first step, data were segmented into epochs starting 1 s before the TMS pulse and ending 1 s after it. We first removed and then replaced data, using a cubic interpolation, from 1 ms before to 10 ms after the TMS pulse from each trial. Afterwards, data were downsampled to 1000 Hz and bandpass filtered between 1 and 80 Hz (Butterworth zero-phase filters). A 50 Hz notch filter was applied to reduce noise from electrical sources. Then, all the epochs were visually inspected and those with excessively noisy EEG were excluded from the analysis. Independent component analysis (INFOMAX-ICA) was applied to the EEG signal to identify and remove components reflecting muscle activity, eye movements, blink-related activity, and residual TMS-related artefacts based on previously established criteria^81,82^. Finally, the signal was re-referenced to the average signal of all the electrodes.

To evaluate changes in cortical excitability we evaluated TEP amplitude locally to the stimulated site. For each participant and for the three stimulation sites (l-DLPFC, r-DLPFC, vertex), we determined the first six peaks through an accurate visual inspection of the TEP waveform: P1 from 15 to 25 ms, P2 from 26 to 47 ms, P3 from 48 to 65 ms, P4 from 66 to 75 ms, P5 from 76 to 115 ms, P6 from 116 to 145 ms. Then, we computed the mean TEP amplitude within each time window within the following electrode clusters: F1, F3, FC1 for l-DLPFC; F2, F4, FC2 for r-DLPFC; C1, C2, CZ for vertex.

Cortical oscillations analysis was performed using a time/frequency decomposition based on Morlet wavelet (cycles = 3; frequency resolution = 1 Hz from 4 to 50 Hz; temporal resolution = 1 ms) and then by computing TMS-related spectral perturbation (TRSP)^83,84^.

To measure oscillations power and to perform the peak shifting analysis we assessed the local TRSP for each stimulated site and stimulation condition. For each TMS area, we considered a pooling computed around the stimulation site (Same as Cortical excitability analysis, see above), and averaged the TRSP values for each of the 23 frequency layers, between 10 and 250 ms for alpha (α) and θ bands, and between 10 and 100 ms for beta (β) and γ band. These time windows were chosen considering the meantime windows of activity.

Wavelet Phase-locking value analysis is a measure of the phase synchronization of a pair of channels. It computes the randomness of the phase-locking between two channels: the index is calculated in a range between 0 and 1, where 0 represents the complete absence of phase-locking and 1 is the total phase synchronization between channels. After the computation of the wavelets and TRSP (see the paragraphs above) we applied the formula to compute the Wavelet Phase Locking Value (W-PLV) as reported in previous studies^85^.

### Statistical analysis

Statistical analyses were performed with SPSS (IBM Corp. Released 2013. IBM SPSS Statistics for Windows, Version 22.0. Armonk, NY: IBM Corp; https://www.ibm.com/analytics/spss-statistics-software). Prior to parametric ANOVAs we assessed the normal distribution of residuals for each variable and assumption of sphericity. If the assumption of sphericity was violated, i.e. Mauchly test *p* < 0.05, we used the Huyhn-Feldt correction. To assess the effect of iTBS–tACS on cortical oscillations, a repeated-measures ANOVA with within-subject factors tACS (γ, θ, sham) and time (T0, T1, T2). For the individual frequency shifting analysis, we calculated the individual frequency peaks (the most expressed frequency in the whole oscillation spectrum) and, equally to the gamma band power analysis, we used a repeated-measures ANOVA with equivalent factors to assess the changes in the band’s expression in terms of shifting.

To assess the effect of iTBS–tACS on cortical excitability, we used a repeated-measures ANOVA with factors tACS, time and peak (P1, P2, P3, P4, P5, P6) with the amplitude of each peak as dependent variable. Also, this analysis was performed separately for each site.

To assess the effect of iTBS–tACS on cortical connectivity, we used a repeated-measures ANOVA with factors electrode (F3 vs. F5; F3 vs. F2) and Time with the mean W-PLV as a dependent variable. All the ANOVAs were performed for each site investigated with TMS-EEG, i.e. l-DLPFC, r-DLPFC and vertex. Post-hoc comparisons were performed with paired t-tests corrected with Bonferroni method. We additionally performed Bayesian statistical analysis on both power gamma cortical oscillations and W-PLV. We performed the analysis using JASP (*JASP Team (2022). JASP (Version 0.8.5.1 [Computer software]*). For what concern the power analysis we performed a two-way Bayesian repeated measures ANOVA (RM-ANOVA) with “tACS” (γ, θ, sham) and “time” (T0, T2) as factors of analysis. For what regard W-PLV we performed a two-way Bayesian repeated measures ANOVA (RM-ANOVA) with “electrode” (F5, F2) and “time” (T0, T1, T2) as factors of analysis.

To assess possible linear relationships between the baseline level of cortical excitability and the effects of tACS-iTBS on cortical oscillations, we performed a correlation analysis between the TEP amplitude recorded at T0, as a measure of cortical excitability, and the difference in the ERSP and PLV values between T2 and T0. This analysis was computed using the Spearman coefficient and was performed for the three iTBS–tACS protocol (iTBS–θtACS, iTBS–γtACS and iTBS–sham tACS).

To assess the predictive value of the baseline level of cortical excitability on the effects of iTBS–tACS in cortico-cortical connectivity, we performed a simple linear regression using the difference in PLV values (T2–T0) as a dependent variable and the TEP amplitude (T0) as a predictor.

## Data Availability

Data could be requested from the corresponding author upon a rational statement.

## Abbreviations

iTBS: Intermittent theta burst stimulation
tACS: Transcranial alternating current stimulation
DLPFC: Dorsolateral prefrontal cortex
TEPs: TMS-evoked potential
TRSP: TMS-related spectral perturbation
W-PLV: Wavelet phase locking value
